# Technical aspects of percutaneous endovascular arteriovenous fistula creation with the Ellipsys® Vascular Access System. Preliminary results after 16 patients

**DOI:** 10.1007/s00423-023-02812-9

**Published:** 2023-02-15

**Authors:** Dominik Liebetrau, Sebastian Zerwes, Hagen Kerndl, Jochen Schaal, Alexander Hyhlik-Dürr

**Affiliations:** Vascular Surgery, Medical Faculty, University of Augsburg, Stenglinstrasse 2, 86156 Augsburg, Germany

**Keywords:** Endovascular vascular access system, Ellipsys**®** catheter, Gracz fistula, Technical aspects, Ellipsys® Vascular Access System

## Abstract

**Purpose:**

Technical aspects are crucial for planning and performing endovascular arteriovenous fistula (AVF) creation. The Ellipsys® Vascular Access System represents a minimal invasive method for the creation of a proximal forearm fistula. This report summarizes the essential elements for AVF creation with the Ellipsys® Vascular Access System and investigates feasibility, efficacy, and safety procedures conducted on 16 patients.

**Materials and methods:**

We performed a retrospective analysis of patients who underwent endovascular AVF creation with the Ellipsys® Vascular Access System between May 2020 and March 2022 at a tertiary referral center.

**Results:**

The median age was 67.5 years (47–86 years). The mean BMI was 31.4 kg/m2. AV fistula was created on 15/16 patients on their left arm. The technical success was 100%. The mean operation time was 24.2 min. There were no complications associated with the procedure. All patients were examined after 30 days (± 5 days). Primary patency after 30 days was 94% (15/16). The mean fistula flow was 681.1 mL/min and the mean AVF diameter was 6.1 mm. Thirteen out of 15 patients met the criteria for potential hemodialysis.

**Conclusion:**

With the Ellipsys Vascular Access System exist an additional possibility of an AV fistula creation. Based on above findings, the Ellipsys® Vascular Access System represents a feasible, safe, and effective method for AVF creation.

## Introduction

**Fig. 1:**
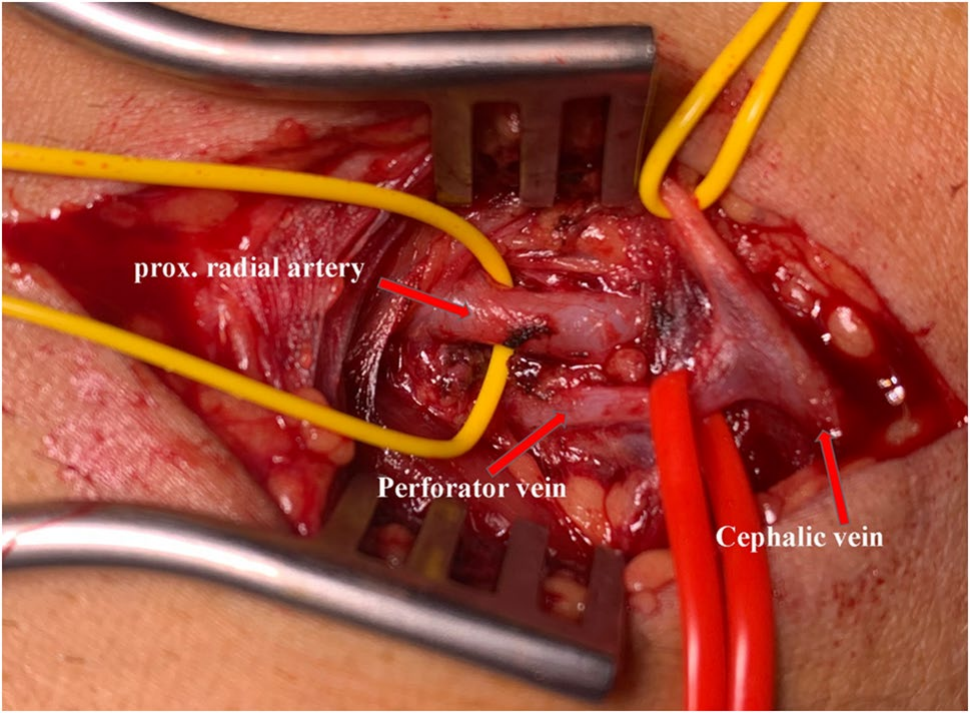
Anatomical proximity between the perforator vein and the prox. radial artery

The arteriovenous fistula (AVF) is the lifeline for patients with end-stage renal disease. These patients are dependent on obtaining safe and functional vascular access for hemodialysis. In the course of the further development of endovascular techniques, it is now possible to create an AVF without open surgical procedures. Since the first description of the Cimino-Brescia fistula in 1966 [1], the principle of the anastomosis technique between vein and artery has not changed. The local venous and arterial conditions determine the localization of AVF. In cases of poor vein and arterial conditions in the wrist, many treating colleagues considered that the next step should be the creation of a proximal arterio-venous fistula (PAV) between the brachial artery (A) and basilic vein or cephalic vein. The possibility of a Gracz fistula [2] (GF) is often disregarded. The GF (perforator vein (PV)/proximal radial artery (PRA)) offers some advantages over PAV (brachial artery/basilic and/or cephalic vein), e.g., longer puncture area, reduction of the risk of hyperdynamic AVF, steal syndrome, and cardiac decompensation [3, 4]. The anatomical proximity between the radial artery and perforator vein **(**Fig. 1**)** offers optimal conditions for the creation of endovascular AVF. Further advantages of the procedure are a constant and reproducible anastomosis, avoidance of scars, and vascular trauma as well as very short intervention times [5–7].

## Objective of the study

Technical aspects are crucial for planning and performing endovascular arteriovenous fistula (AVF) creation. The Ellipsys® Vascular Access System represents a minimal invasive method for the creation of a proximal forearm fistula. This report summarizes the essential elements for AVF creation with the Ellipsys® Vascular Access System and investigates feasibility, efficacy, and safety measures conducted on 16 patients.

## Patients and methods

Between May 1, 2019 and March 15, 2022, all patients underwent AVF by Ellipsys**®** Vascular Access at tertiary referral center.

Informed consent was obtained from all patients. The Ludwig-Maximilians-University Munich (LMU) ethics committee peer reviewed the project (22–0432).

The results were prospectively recorded in a database (Microsoft Excel® version 2019) and analyzed retrospectively. Sixteen patients (13 male, 3 female) with pre- or end-stage renal disease were included. Perioperative complications have been defined as hand ischemia, bleeding, and infections. The technical success was defined as the successful completion of the procedure, as well as the intraoperative control of the AVF flow by ultrasound. A follow-up exam was performed after 30 days (± 5 days). The AVF was re-examined clinically and by ultrasound to evaluate a successful maturation. Maturation was defined as a brachial artery blood flow of ≥ 500 mL/min with an AVF diameter of ≥ 5 mm.

## Description of the Ellipsys® Vascular Access System

**Fig. 2:**
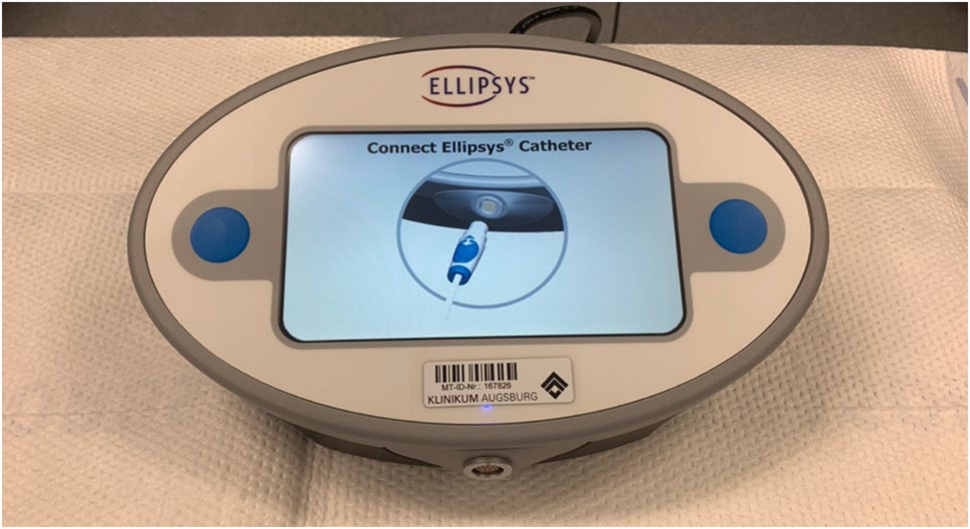
Ellipsys® power controller

**Fig. 3:**
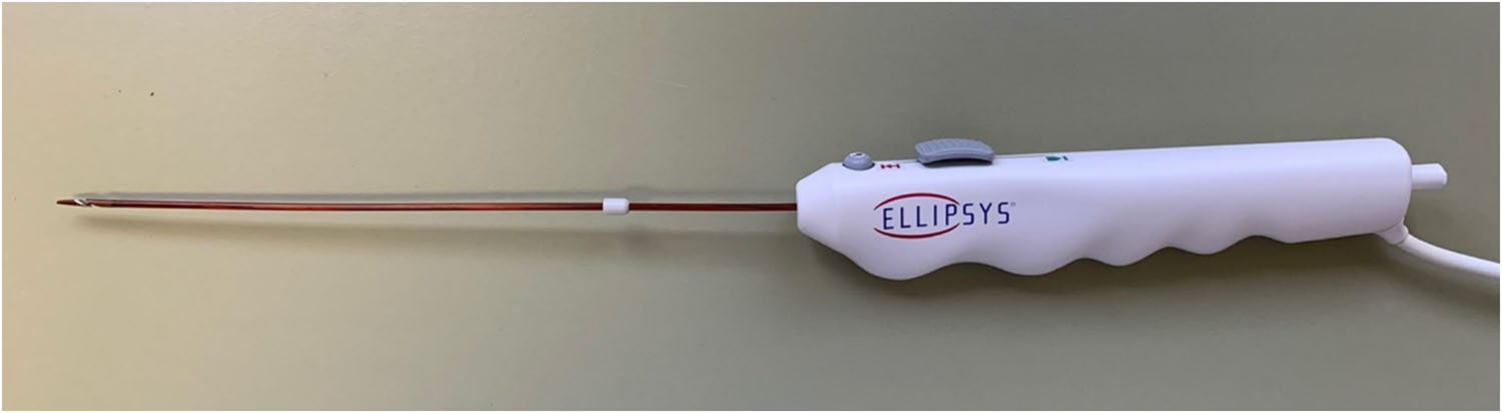
Ellipsys ® catheter

The Ellipsys**®** Vascular Access System is a thermal resistance anastomosis device (TRAD) for the minimally invasive creation of an anastomosis (side to side) between the proximal radial artery and the perforator vein. The device establishes a tissue fuse anastomosis with an immediate and permanent connection between the artery and vein [8]. The catheter requires a 6-French access (e.g., 6 Fr. Glidesheath Slender, Terumo Medical Corporation, NJ 08873) and is connected to a generator (Fig. 2). The catheter is divided into a long shaft area with a tip (responsible for the creation of the anastomosis) and a handle with a slider (Fig. 3). This is used to open and close the catheter tip.

## Anatomical criteria

For the evaluation of any AVF procedures, a standardized, ultrasound-guided vein mapping is executed for each patient. An endovascular AVF creation takes place only after exhaustion of further distal (e.g., radiocephalic) methods in the forearm.

The Ellipsys**®** Vascular Access System can be used under the following anatomical conditions:

1. Ø median cubital vein/basilic vein and/or cephalic vein ≥ 2 mm
2. Ø proximal radial artery ≥ 2 mm
3. 3 Ø perforator vein ≥ 2 mm
4. distance perforator vein to proximal radial artery ≤ 1.5 mm

For preoperative planning, other factors need to be taken into account such as the angle of the perforator vein to the superficial access veins. If the angle between the perforator vein and the median cubital vein or cephalic vein is very steep, an endovascular AVF creation can fail, despite the anatomical requirements being met.

Even a deep crossing point between the veins and arteries or a curved course of the perforator vein can negatively affect the feasibility of AVF creation. Ideal conditions for a successful AVF creation can be observed in the video “Screening.”

## Basic knowledge

The animation explains the basics of the procedure. The key to a successful procedure is the handling of the ultrasound device. Knowing the current position of the needle tip in the vessel is the most important thing. In particular, the coordination between the ultrasound transducer and the navigation of the needle requires very good sonographic understanding. By accurately repositioning the ultrasound transducer, the optimal image quality can be achieved and the needle tip can be navigated successfully.

**Fig. 4:**
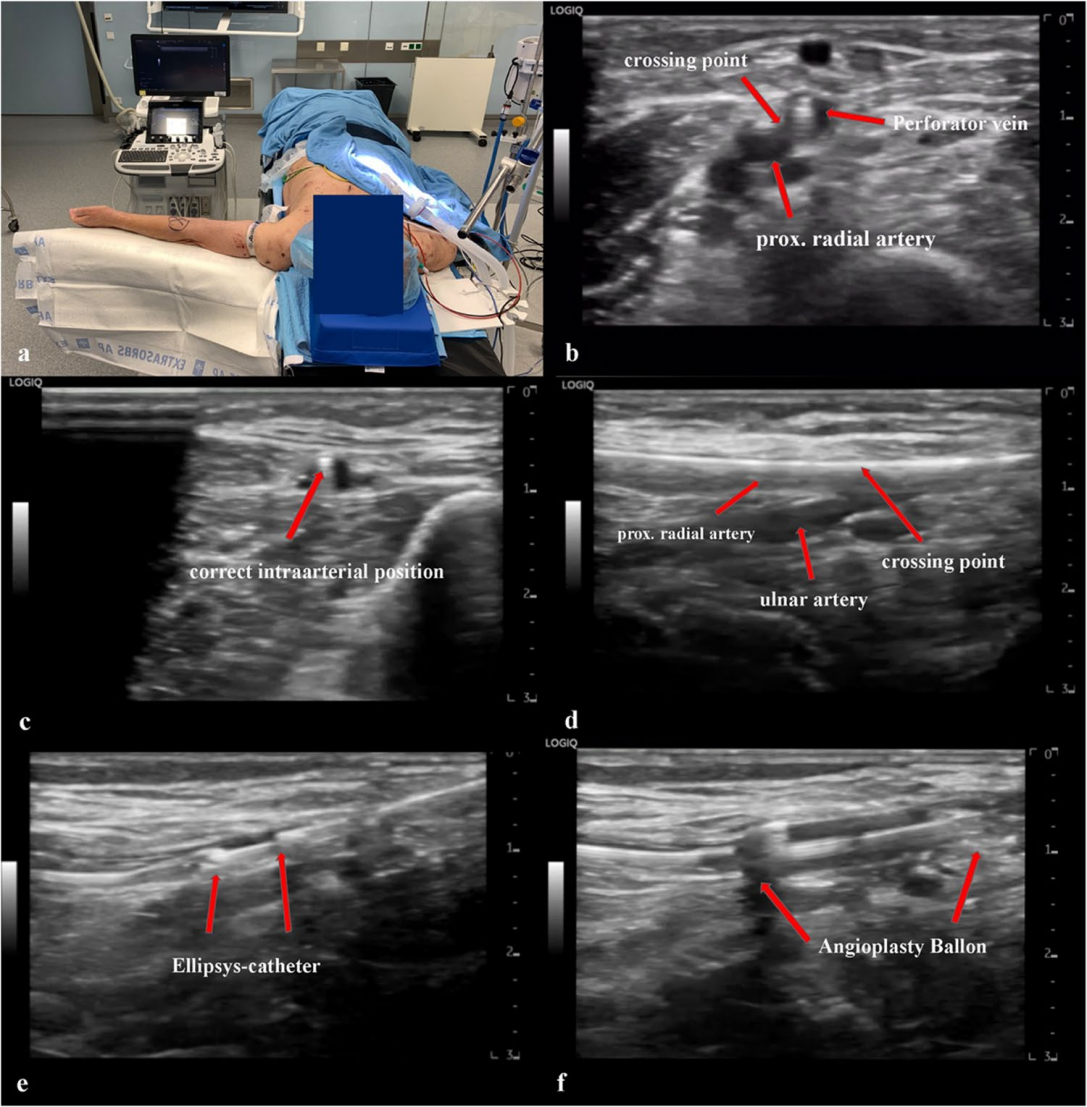
Overview of the procedure: **a** preoperative positioning and positioning of the ultrasound device; **b** ultrasound cross-section, proximal forearm, showing the crossing point between the perforating vein and the proximal radial artery; **c** ultrasound cross-section forearm, checking the guidewire for correct intra-arterial position. The arrow marks the guidewire (anechoic point); **d** ultrasound longitudinal section prox. forearm, visualization of the brachialis bifurcation with correct intra-arterial position of the guidewire; **e** ultrasound longitudinal section prox. forearm, positioning of the Ellipsys catheter; **f** ultrasound longitudinal section prox. forearm, PTA of the anastomosis using a 5 × 20 mm monorail balloon after successful application of the Ellipsys catheter

After successfully puncturing the artery and the verification of the correct position of the guide wire (Fig. 4c, d), the procedure can usually be completed easily. Intraoperatively, no X-rays or contrast agents are necessary.

Before using the procedure for the first time, we recommend intensive training on the model to learn the coordination between the ultrasound transducer and the needle tip.

## Operational setting

**Fig. 5:**
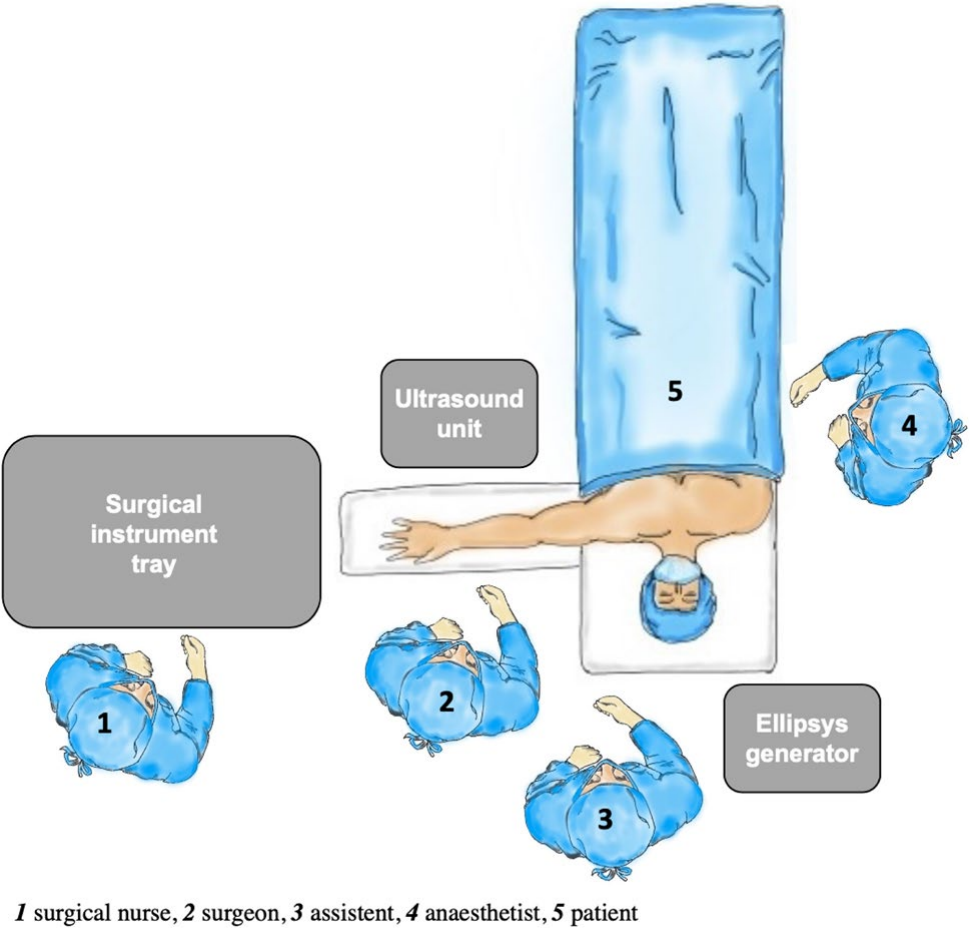
Preoperative setting left arm

**Fig. 6:**
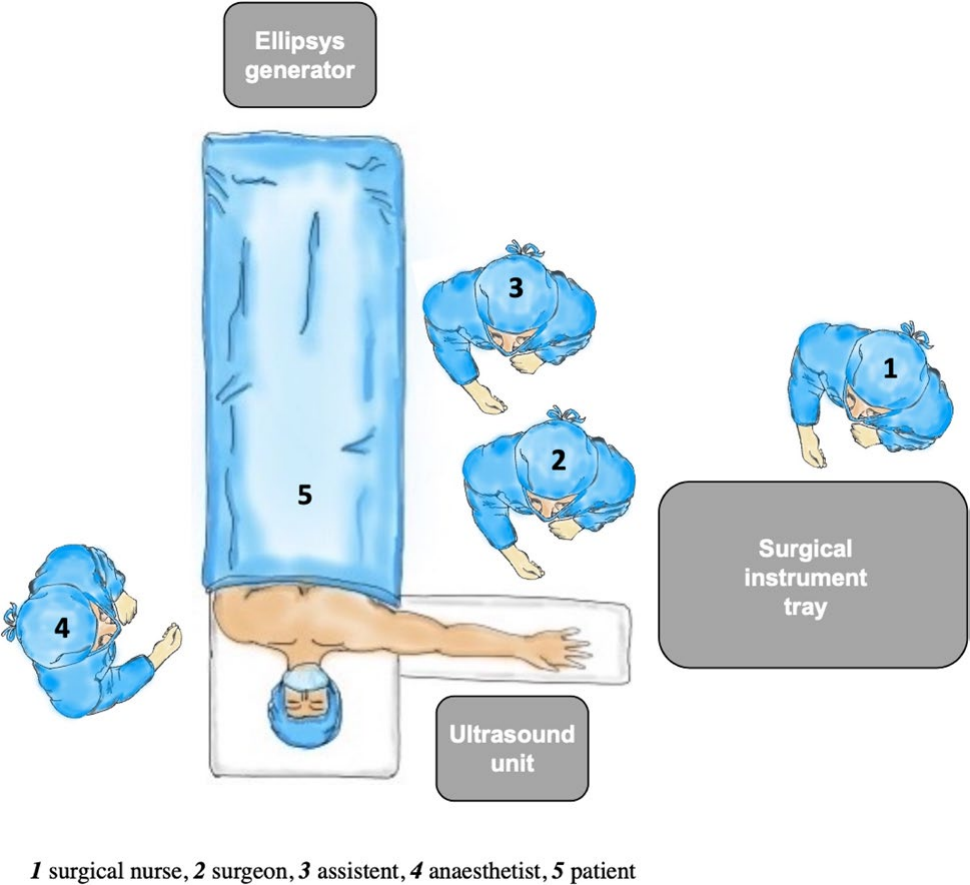
Preoperative setting right arm

The procedure is performed in supine position and under plexus anesthesia. The arm, which is designated for the AFV creation, is placed on a side table with the palm facing upward. Before sterile washing, a tourniquet is attached to the proximal upper arm. The ultrasound device is placed opposite the surgeon (Fig. 4a). The preoperative setting is shown in Figs. 5 and 6.

## Anticoagulation

Preoperative loading using acetylsalicylic acid (ASA) and clopidogrel is not administered. To avoid thrombosis and early failure, intraoperative anticoagulation is necessary. When the artery has been successfully punctured and the correct position of the guide wire has been established (Fig. 4c, d), a single dose of 3000 international units of heparin is administered. A dose adjustment can be made, e.g., with increased body mass index. Due to single administration and short intervention times, there is no intraoperative monitoring (e.g., activated clotting time (ACT) determination) executed.

## Access vessel

The superficial veins are chosen for access. Depending on the anatomy (see above), the median cubital vein or the cephalic vein can be punctured about 1–2 cm before the perforator vein is separating.

## Materials

The following materials are required:

- 1 × Ellipsys.**®** Catheter (Medtronic, 710 Medtronic Parkway Minneapolis)
- 1 × Ellipsys.**®** Generator (Medtronic, 710 Medtronic Parkway Minneapolis)
- 1 × Ellipsys.**®** Access Kit, consisting of 6 Fr. “slender sheath” (reduced outer diameter), micropuncture needle, and 0.018-in guidewire (Medtronic, 710 Medtronic Parkway Minneapolis)
- 1 × 0.014-in guidewire for the Ellipsys.**®** catheter (e.g., Nitrex 0.014, 80 cm, Medtronic, 710 Medtronic Parkway Minneapolis)
- 1 × 5 × 20 mm Monorail Balloon (e.g., Sterling Monorail Ballon 5 × 20 mm, Boston Scientific, Marlborough, MA 01752 (USA))
- 1 × manometer (e.g., Encore Inflation Device Boston Scientific, Marlborough, MA 01752 (USA))

## Description of the procedure

After the disinfection and sterilization of the arm, the anatomical conditions are examined again by ultrasound. The puncture should be done by a micropuncture needle. We recommend an access of about 1 to 2 cm max. above the division of the perforator vein. This allows the procedure to be successful despite the perforator vein being at an unfavorable angle in comparison to the superficial veins. Subsequently, the tip of the needle must be positioned centrally in the vessel with the aid of an ultrasound (hyperechoic point in the vessel (Fig. 4b) after which the needle is gradually advanced under sonographic control. The tip of the needle is navigated from the superficial arm vein into the perforator vein. After the needle has been positioned in the perforator vein, the crossing point to the proximal radial artery must be found, which may not exceed 1.5 mm (Fig. 4b). Depending on the position of the perforator vein, the proximal radial artery can then be punctured. After the successful verification of the correct intraarterial needle tip position (sonographic control in transverse and longitudinal sections) (Fig. 4c, d), a 0.018-in guidewire is inserted into the radial artery. Subsequently, 3000 international units of heparin is intravenously administered to the patients. The next step is to remove the micropuncture needle and a 6 Fr. a slender sheath (reduced outer diameter) is inserted into the radial artery via the 0.018-inch guidewire.

The introducer is left inside and the wire located over the introducer is exchanged for a 0.014-in wire. Once the exchange is made, the introducer is removed. The next step is for the Ellipsys**®** catheter to be inserted, connected to the generator, and to be positioned under sonographic control. It is important that the catheter tip is placed into the radial artery and that the catheter base can be inserted in the perforator vein (Fig. 4e). Once the correct position is successfully verified, the Ellipsys**®** catheter can be closed and the distance between the artery and vein can now be read off the generator. A distance of 0 mm would be an indication that the arterial and venous vascular wall is not located between the Ellipsys**®** catheter. Standard values are between 0.1 and 0.9 mm. After verifying the distance, the catheter is activated and a tissue-fused permanent anastomosis is created. The Ellipsys**®** catheter can then be removed. After execution of the Ellipsys catheter, a palpable thrill can be noticed.

To increase the primary patency, a simultaneous angioplasty of the anastomosis is recommended. The arteriovenous anastomosis is dilated for at least 90 s **(**Fig. 4f**)** with a 5 × 20 mm monorail balloon. The deep venous system usually remains untouched. Thereafter, the materials are removed and the puncture is gently pressed off for 3–5 min. In addition, the puncture site is secured with steri-strips and covered by a sterile plaster.

## Follow-up protocol

Four weeks after the intervention procedure, a follow-up is undergone whereby the AVF will be investigated via ultrasound and clinical examination. At the first follow-up, the maturation of the AVF can already be estimated. If necessary, additional maturation procedures (angioplasty, banding, etc.) can be planned. The aim is an AVF blood flow of at least 500 mL/min and a minimum diameter of 5 mm. Further follow-up examinations are planned after 3, 6, and 12 months, respectively, after the intervention procedure.

## Results

The median age was 67.5 years (47–86 years) with a mean body mass index of 31.4 kg/m^2^. The 30-day mortality rate was 0%. Diabetic nephropathy (9/16) was the most common reason for renal insufficiency (NI). AVF was performed in 15/16 cases on the left arm. Nine out of 16 patients had end-stage renal disease and were dialyzed via a central venous catheter. Six out of 16 patients already had a previous AVF on the same arm **(**Table 1**)**. All patients met the anatomical criteria for the Ellipsys**®** Vascular Access System procedure. The mean anatomical diameter of the proximal radial artery was 3.1 ± 1.2 mm, perforator vein 2.7 ± 0.8 mm, and cephalic vein 3.4 ± 1.1 mm. The mean distance between perforator vein and artery was 1.1 ± 0.2 mm **(**Table 2). Technical success was 100%. The mean operation time was 24.2 min. In 15/16 cases the cephalic vein was chosen for vascular access. In 14 cases, the anastomosis was created between the proximal radial artery and the perforator vein and in 2 cases between the brachial artery and the perforator vein. No perioperative complications were observed (Table 3).

**Table 1:** Patient characteristics

	Overall	
	*n*	%
Included patients	16	100.0
Sex (m/f)	13|3	81.3|18.7
Age in years (mean) BMI^1^	67.5 (47–86)	
	31.4 ± 9.3	
Hypertension	15	93.8
Diabetes	9	56.3
Coronary heart disease	11	68.8
Reason for renal failure		
Diabetic nephropathy	9	56.3
Atrophic kidney	2	12.5
Endocarditis	1	6.3
ANCA-associated vasculitis	1	6.3
Other	3	18.8
Renal disease		
Terminal	9	56.2
Preterminal	7	43.8
Dialysis via central venous catheter^2^	9	60.0
Former arteriovenous fistula on the same arm	6	40.0

^1^Body mass index; ^2^ at time of procedure

**Table 2:** Anatomical conditions

		Overall	
		*n*	%
Included patients		16	100.0
Diameter [mm]			
Brachial artery		4.3 ± 1.2	
Proximal radial artery		3.1 ± 1.2	
Perforator vein		2.7 ± 0.8	
Distance perforator vein to artery		1.1 ± 0.2	
Cephalic vein		3.4 ± 1.1	
Basilic vein		3.4 ± 1.7

**Table 3:** Procedure

	Overall	
	*n*	%
Included patients	16	100.0
Technical success [%]	100.0	
Procedure time [minutes (min–max)]	24.2 (13–54)	
Side (left/ right)	15|1	
Anastomosis		
Brachial artery	2	13.3
Radial artery	14	86.7
Vascular access		
Cephalic vein	15	93.8
Median cubital vein	1	6.3
30-day mortality	0	0.0
Complications^1^	0	0.0

^1^Such as ischemia, bleeding, and infection

The primary patency after 30 days was 94% (15/16) with a mean AVF blood flow of 681.1 mL/min and a mean AFV diameter of 6.1 mm. Thirteen out of 16 patients met the criteria for maturation (Table 4).

**Table 4:** Follow-up after 4 weeks

		Overall	
		*n*	%
Included patients		16	100.0
patency		15	94.0
Fistula blood flow [mL/min]^2^		681.1 ± 183.4	
Outflow vein			
Cephalic vein		8	53.3
Basilic vein		2	13.1
Cepahalic and basilic veins		3	20
Brachial vein		2	13.3
Diameter outflow vein [mm]		6.1 ± 1.2	
Met criteria for dialysis^1^		13/16	86.7

^1^Fistula flow ≥ 500 mL/min and diameter vein ≥ 5 mm; ^2^ measured by brachial artery blood flow

## Discussion

The Ellipsys**®** Vascular Access System represents a safe and effective (technical success 100%) means of creating an access for hemodialysis. The procedure is complex and the learning curve can possibly be flattened through systematic training.

The age range was from 47 to 86 years, with a median age of 67.5 years and the gender distribution was unbalanced with 13 patients being male and 3 patients being female. The mean body mass index was 31.4 kg/m^2^. The most common reason for hemodialysis was diabetic nephropathy (Table 1). This correlates with the data from the literature sources [9, 10]. In all patients, the AVF procedure was performed on the non-prominent arm. The mean operation time was 24.2 min. An isolated analysis of the last 10 procedures performed shows a mean operation time of 15.9 min (learning curve effects). These results are consistent with the data from the literature sources [3, 7]. A learning curve of 5–10 cases is also described in the literature source [11], but is dependent on personal sonographic skills.

The technical success was 100% and correlates with the data from the literature sources [3, 6, 12]. The correct puncture of the proximal radial artery is the key to a successful procedure, and the position and direction of the needle must be visible at all times. This requires the permanent correction of the ultrasound transducer for the accurate localization and navigation of the needle tip. These skills must be learned, and for this reason, we favor structured training with the “Ellipsys**®** Vascular Access System.”

In addition to ultrasound skills, technical success depends on the correct assessment of the anatomical conditions. The fulfillment of the anatomical criteria does not guarantee a completely successful implementation (refer to anatomical criteria).

The evaluation is subject to empirical values ​​and thus also to a learning curve; therefore, previous training can speed up this process.

Perioperative complications did not occur overall **(**Table 3). This is consistent with the literature source [3], thus, proving that when the Ellipsys**®** Vascular Access System is performed in the hands of an experienced user, it is a safe and effective procedure [12, 13].

Potentially, vascular perforations, embolisms, and hand ischemia can occur as a result of the procedure. Redoing procedures for successful maturation (e.g., angioplasty, banding) may be necessary. Therefore, expertise in dealing with AV fistulas is required. The primary patency after 30 days was 94% (15/16) with 1 patient presenting an occlusion of arterial anastomosis. Because no side branches are ligated during the application of the Ellipsys**®** Vascular Access System and the deep brachial vein is not coiled, the original anatomical conditions are still present in case of an early closure. In principle, the procedure can be repeated. In this case, an open-surgical AVF was created without complications. Complementary maturation procedures have been reported by Mallios et al. [3].

The biggest advantage of the Ellipsys Vascular Access System is the additional option to create an AV fistula. Other advantages compared to the surgical installation are the very fast procedure time, the creation of a constant, reproducible and user-independent anastomosis, and the fact that there is no dissection of the vein (CAVE: anastomotic stenosis) [3, 4, 7]. Furthermore, no foreign material remains in the patient. One point patients should be made aware of is the fact that percutaneous AV fistulas probably require a higher rate of maturation procedures [14]. Once the fistula is maturated, the number of interventions per patient/year is low [14]. In a comparison of surgical vs. percutaneously created AV fistulas, the material use (catheter price approx. $5000) must be regarded as critical. Nonetheless, every possibility to create a functional AV fistula should be taken.

Finally, the Ellipsys® Vascular Access System should be performed by a user experienced in AV fistula management.

The structured learning of the procedure is integral to the safe and effective use of the Ellipsys**®** Vascular Access System. With the Ellipsys**®** Vascular Access System, additional possibilities for AVF creation are made possible; however, further research is needed in this regard.

## Conclusion

For a safe implementation of the Ellipsys**®** Vascular Access System, intensive training/coaching is required to learn the coordination between the ultrasound transducer and the navigation of the needle. Technical success depends on the correct assessment of anatomical conditions—CAVE: fulfillment of the anatomical criteria does not equate to successful implementation. The Ellipsys® Vascular Access System should be performed by a user experienced in AV fistula management. Our experience to date and the studies available show a safe and functional vascular access.

## References

[1] Brescia MJ, Cimino JE, Appel K (1966). Chronic hemodialysis using venipuncture and a surgically created arteriovenous fistula. N Engl J Med.

[2] Gracz KC, Ing TS, Soung LS (1977). Proximal forearm fistula for maintenance hemodialysis. Kidney Int.

[3] Mallios A, Bourquelot P, Franco G (2020). Midterm results of percutaneous arteriovenous fistula creation with the Ellipsys Vascular Access System, technical recommendations, and an algorithm for maintenance. J Vasc Surg.

[4] Mallios A, Fonkoua H, Allouache M (2020). Percutaneous arteriovenous dialysis fistula. J Vasc Surg.

[5] Hull JE, Jennings WC, Cooper RI (2018). The pivotal multicenter trial of ultrasound-guided percutaneous arteriovenous fistula creation for hemodialysis access. J Vasc Interv Radiol.

[6] Shahverdyan R, Beathard G, Mushtaq N (2020). Comparison of outcomes of percutaneous arteriovenous fistulae creation by ellipsys and wavelinq devices. J Vasc Interv Radiol.

[7] Shahverdyan R, Beathard G, Mushtaq N (2021). Comparison of ellipsys percutaneous and proximal forearm Gracz-type surgical arteriovenous fistulas. Am J Kidney Dis.

[8] Cezo JD, Kramer E, Taylor KD (2013). Temperature measurement methods during direct heat arterial tissue fusion. IEEE Trans Biomed Eng.

[9] Ramanathan AK, Nowacki J, Hoffman H (2012). An ode to Cimino. J Vasc Access.

[10] Shahverdyan R, Tabbi P, Mestres G (2022). Multicenter European real-world utilization of VasQ anastomotic external support device for arteriovenous fistulae. J Vasc Surg.

[11] Isaak A, Mallios A, Gurke L (2020). Teleproctoring in vascular surgery to defy COVID-19 travel restrictions. Eur J Vasc Endovasc Surg.

[12] Hebibi H, Achiche J, Franco G (2019). Clinical hemodialysis experience with percutaneous arteriovenous fistulas created using the Ellipsys(R) Vascular Access System. Hemodial Int.

[13] Yan Wee IJ, Yap HY, Hsien Ts'ung LT (2019). A systematic review and meta-analysis of drug-coated balloon versus conventional balloon angioplasty for dialysis access stenosis. J Vasc Surg.

[14] Hull JE, Jennings WC, Cooper RI (2022). Long-term results from the pivotal multicenter trial of ultrasound-guided percutaneous arteriovenous fistula creation for hemodialysis access. J Vasc Interv Radiol.

